# Prognostic Values of EPDR1 Hypermethylation and Its Inhibitory Function on Tumor Invasion in Colorectal Cancer

**DOI:** 10.3390/cancers10100393

**Published:** 2018-10-22

**Authors:** Chun-Ho Chu, Shih-Ching Chang, Hsiu-Hua Wang, Shung-Haur Yang, Kuo-Chu Lai, Te-Chang Lee

**Affiliations:** 1 Institute of Pharmacology, National Yang-Ming University, Taipei 11221, Taiwan; chchu@kfsyscc.org; 2 Department of Surgery, Koo Foundation, Sun Yat-Sen Cancer Center, Taipei 11259, Taiwan; 3 Division of Colon & Rectal Surgery, Department of Surgery, Taipei Veterans General Hospital, Taipei 11217, Taiwan; changsc@vghtpe.gov.tw; 4 Institute of Biomedical Sciences, Academia Sinica, Taipei 11529, Taiwan; qqsunny_82@hotmail.com; 5 Department of Surgery, National Yang-Ming University Hospital, Yilan 26058, Taiwan; yangsh@vghtpe.gov.tw; 6 Department of Pharmacology, Tzu Chi University, Hualien 97004, Taiwan

**Keywords:** CRC, MSI, *EPDR1* methylation, prognosis, invasion

## Abstract

Aberrant DNA methylation is a potential mechanism underlying the development of colorectal cancer (CRC). Thus, identification of prognostic DNA methylation markers and understanding the related molecular functions may offer a new perspective on CRC pathogenesis. To that end, we explored DNA methylation profile changes in CRC subtypes based on the microsatellite instability (MSI) status through genome-wide DNA methylation profiling analysis. Of 34 altered genes, three hypermethylated (epidermal growth factor, *EGF*; carbohydrate sulfotransferase 10, *CHST10*; ependymin related 1, *EPDR1*) and two hypomethylated (bone marrow stromal antigen 2, *BST2*; Rac family small GTPase 3, *RAC3*) candidates were further validated in CRC patients. Based on quantitative methylation-specific polymerase chain reaction (Q-MSP), *EGF*, *CHST10* and *EPDR1* showed higher hypermethylated levels in CRC tissues than those in adjacent normal tissues, whereas *BST2* showed hypomethylation in CRC tissues relative to adjacent normal tissues. Additionally, among 75 CRC patients, hypermethylation of *CHST10* and *EPDR1* was significantly correlated with the MSI status and a better prognosis. Moreover, *EPDR1* hypermethylation was significantly correlated with node negativity and a lower tumor stage as well as with mutations in B-Raf proto-oncogene serine/threonine kinase *(BRAF*) and human transforming growth factor beta receptor 2 (*TGFβR2*). Conversely, a negative correlation between the mRNA expression and methylation levels of *EPDR1* in CRC tissues and cell lines was observed, revealing that DNA methylation has a crucial function in modulating *EPDR1* expression in CRC cells. *EPDR1* knockdown by a transient small interfering RNA significantly suppressed invasion by CRC cells, suggesting that decreased EPDR1 levels may attenuate CRC cell invasion. These results suggest that DNA methylation-mediated *EPDR1* epigenetic silencing may play an important role in preventing CRC progression.

## 1. Introduction

Colorectal cancer (CRC) is the third most common cancer and fourth leading cause of cancer death worldwide. In 2016, an estimated 134,490 cases of CRC were diagnosed, with 49,190 deaths in the United States [1]. The risks for CRC include age, obesity, physical inactivity, smoking, alcohol drinking, high consumption of red or processed meat, low calcium intake, and very low intake of fruit and vegetables [1]. Additionally, hereditary factors include a family history of CRC and/or polyps, Lynch syndrome, and a personal history of chronic inflammatory bowel disease [2]. Common treatments for CRC include surgery, radiation, and chemotherapy. However, the 5- and 10-year survival rates for CRC are 65% and 58%, respectively [2]. Furthermore, approximately 50% of CRC patients will develop liver metastasis during the course of their disease [3], and the 10-year survival rates of these patients are only 17 to 28% [4]. Therefore, early detection and identification of useful diagnostic and prognostic markers are key to increase CRC survival rates.

Evidence accumulated since the 1990s has demonstrated three molecular pathways involved in CRC pathogenesis: chromosomal instability (CIN), microsatellite instability (MSI), and the CpG island methylator phenotype (CIMP). Through the CIN pathway, genetic alterations are generated in tumor suppressor genes (such as adenomatous polyposis coli (*APC*), tumor protein 53 (*TP53*) and SMAD family member 4 (*SMAD4*)) and oncogenes (such as K-ras proto-oncogene, *KRAS* and phosphoinositide 3-kinase catalytic subunit-α (*PI3KCA*)), resulting in CRC development [5,6,7]. Approximately 15% of CRCs present with MSI due to either defective DNA mismatch repair (MMR) induced by a mutation or methylation of an MMR gene (mutL homolog 1, *MLH1*; mutS protein homolog 2, *MSH2*; mutS homolog 6, *MSH6*; or PMS1 homolog 2, *PMS2*) promoter [8,9].

Epigenomic studies have demonstrated that tumors with MSI have a high CIMP phenotype and, hence, exhibit hypermethylation of genes critical for tumor progression [10,11]. *MLH1* methylation is a main event observed in CRC with high CIMP (CIMP-H) [12]. In fact, high MSI (MSI-H) and CIMP-H share similar molecular features because nearly all MSI-H CRCs are molecularly based on hypermethylation-induced silencing of the *MLH1* gene promoter [12]. CRCs with MSI and/or CIMP have distinct clinicopathological features, including the following: a tendency to arise in the proximal colon, lymphocytic infiltration, and a poorly differentiated, mucinous or signet ring appearance [13,14,15,16]. Additionally, these cases have a better prognosis than those without MSI but show no benefit from 5-fluorouracil (5-FU) treatment [17,18,19,20]. Overall, identification of biomarkers for MSI and/or CIMP and understanding the related molecular functions may offer a new perspective about MSI in CRC.

Epigenetic dysregulation of gene expression plays a vital role in the initiation and progression of cancer. DNA methylation is an epigenetic process through which the silencing of gene expression occurs and can be reversed by a DNA-demethylating agent, such as 5-aza-2’-deoxycytidine (5-Azadc). CIMP in CRC is characterized by simultaneous hypermethylation of CpG islands in a subset of genes [10]. To assess CRC CIMP, Weisenberger et al. developed a marker panel of five genes (calcium voltage-gated channel subunit alpha1 G, *CACNA1G*; insulin-like growth factor-2, *IGF2*; neurogenin-1, *NEUROG1*; runt-related transcription factor 3, *RUNX3*; and suppressor of cytokine signaling 1, *SOCS1*) [21]. Additionally, MSI patients with a methylated *MLH1* promoter have high CIMP, another epigenetic feature that is clinically valuable to predict outcomes in CRC patients. Indeed, aberrant DNA methylation is a common and early alteration in many types of human cancer, including CRC [22,23,24]. To date, hypermethylation of the promoter of several genes, including *APC*, *p16INK4a*, tissue inhibitor of metallopeptidase-3 (*TIMP3*), twist-related protein 1 (*TWIST1*), and growth arrest-specific 7 (*GAS7*), has been reported in CRC [25,26], and several DNA methylation markers have been proposed as useful early biomarkers to detect CRC [27,28,29]. Thus, molecular studies aimed at discovering CRC-specific methylation markers may provide useful insight into the molecular mechanisms of CRC progression.

In this study, we explored DNA methylation profile changes in CRC with MSI. Five aberrantly methylated genes (epidermal growth factor, *EGF*; carbohydrate sulfotransferase 10, *CHST10*; ependymin related 1, *EPDR1*; bone marrow stromal antigen 2, *BST2*; and Rac family small GTPase 3, *RAC3*) were further verified in CRC tumor tissues. We investigated the clinical relevance of the methylation status of these five genes in CRC patients. We further demonstrated a negative correlation between the mRNA expression and DNA methylation levels of *EPDR1* in CRC tissues and cell lines, indicating that DNA methylation may have a major function in modulating *EPDR1* expression in CRC cells. Additionally, we explored the inhibitory function on the tumor invasion of *EPDR1* in CRC cells.

## 2. Results

### 2.1. The EGF, CHST10, EPDR1, BST2, and RAC3 Methylation Levels Are Validated in CRC

To delineate DNA methylation profile changes in CRC with MSI, we performed DNA methylation analysis using Infinium Human Methylation 27K BeadChip (Illumina, San Diego, CA, USA). Together with the MSS group, methylation profiles were analyzed using three pooled DNA samples for each group. Consistent with other reports, over 300 CpG loci were hypermethylated in the methylated MLH1 group compared with the other two groups (data not shown). Compared with the MSS group, 650 selected genes in the MSI-MLH1 methylated or MSI-MLH1 mut groups showed an absolute beta difference (Δβ) value more than 0.5 (hypermethylation) or less than −0.25 (hypomethylation) (Figure S1A). Among them, 10 hypermethylated and 24 hypomethylated genes were identified in both MSI groups compared with those in the MSS group (Figure S1B,C).

**Figure 1:**
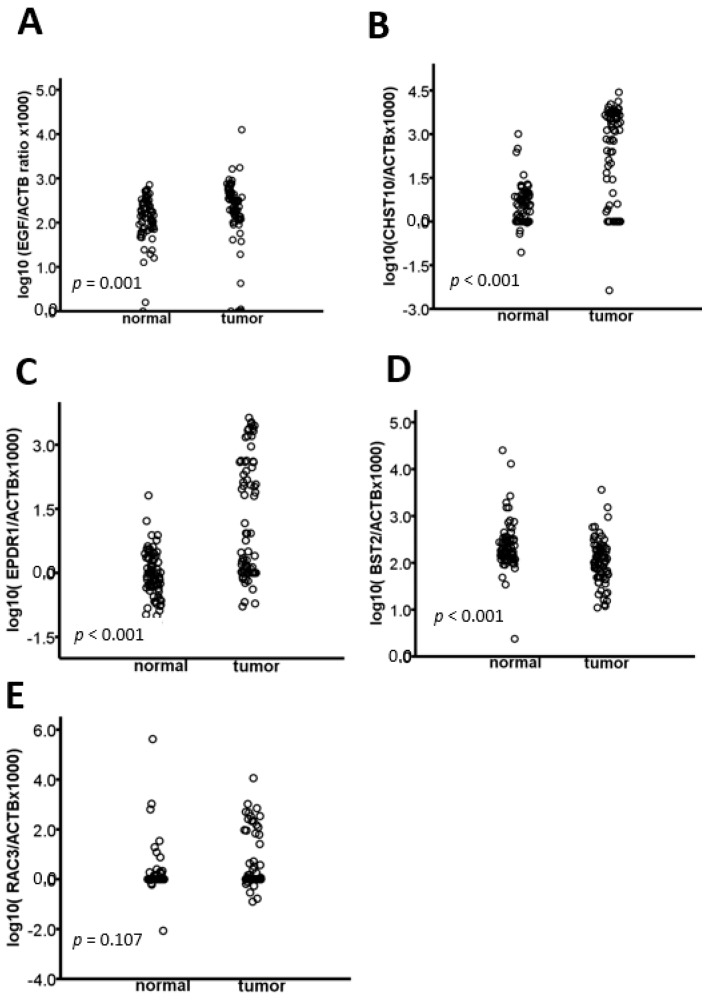
Methylation levels of (**A**) *EGF*, (**B**) *CHST10*, (**C**) *EPDR1*, (**D**) *BST2* and (**E**) *RAC3* in 75 colorectal cancer (CRC) tissues and adjacent non-cancerous tissues, as determined by quantitative methylation-specific polymerase chain reaction (Q-MSP). Normalization to β-actin (*ACTB*) was performed for all genes. *p* values were derived from the Mann-Whitney U test. *EGF*: epidermal growth factor; *CHST10*: carbohydrate sulfotransferase 10; *EPDR1*: ependymin related 1; *BST2*: bone marrow stromal antigen 2; *RAC3*: Rac family small GTPase 3.

Among these hypermethylated candidate genes, *EPDR1* and *EGF* were reported to be deregulated in CRC tissues compared with those in adjacent normal and normal colon tissues [30,31]. *CHST10* is likely a potential methylation biomarker and therapeutic target of vincristine in CRC cells [32]. Regarding the hypomethylated candidate genes, overexpression of *BST2* is associated with poor survival in patients with CRC as well as those with esophageal or gastric cancer [33]. Additionally, the silencing of *RAC3* inhibits proliferation and induces apoptosis in human lung cancer cells [34]. Accordingly, the three hypermethylated (*EGF*, *CHST10*, *EPDR1*) and two hypomethylated (*BST2*, *RAC3*) candidate genes were further validated by Q-MSP using 75 pairs of CRC and adjacent normal tissues. The clinicopathological features of these patients are provided in Table 1. We found that *EGF*, *CHST10* and *EPDR1* showed higher hypermethylated levels in CRC tissues than those in adjacent normal tissues (Figure 1A–C, respectively). Additionally, *BST2* showed hypomethylation in CRC tissues than those in adjacent normal tissues (Figure 1D), although the methylation status of *RAC3* was not significantly different (Figure 1E).

### 2.2. CHST10 and EPDR1 Hypermethylation Is Significantly Correlated with a Better Prognosis

**Figure 2:**
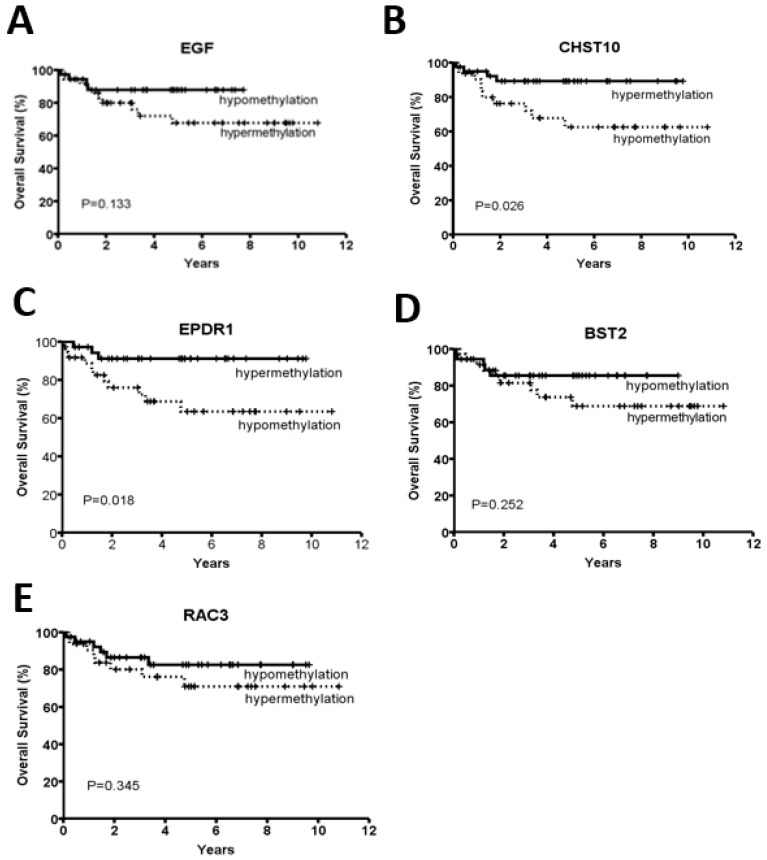
Kaplan-Meier analysis of overall survival in 75 CRC patients according to the methylation status of (**A**) *EGF*, (**B**) *CHST10*, (**C**) *EPDR1*, (**D**) *BST2* and (**E**) *RAC3*. CRC patients were divided into two groups based on the methylation cut-off points of five genes, as described in the Materials and Methods section. *p* values were derived from the log-rank test.

We further analyzed the correlation of the methylation levels of these five candidate genes and various clinicopathological factors, including the differentiation status, invasion depth, node status, tumor stage, and microsatellite status. Compared with MSS CRC patients, associations with a lower median age of incidence (*p* < 0.001), female gender (*p* = 0.004), poor differentiation (*p* = 0.001) and proximal tumor location (*p* < 0.001) were found for patients with MSI CRC (Table 1). Furthermore, the methylation levels of *CHST10* and *EPDR1* were significantly higher in CRC patients with MSI than in those with MSS (*CHST10*: *p* = 0.003; *EPDR1*: *p* < 0.001) (Table 2). By contrast, the *BST2* and *RAC3* methylation levels were significantly lower in CRC patients with MSI than in those with MSS (*BST2*: *p* = 0.015; *RAC3*: *p* < 0.001). Interestingly, *EPDR1* hypermethylation was significantly correlated with node negativity (*p* = 0.044) and an early tumor stage (*p* = 0.044) (Table 2). We also examined the relationship of the methylation status of these five genes with an overall survival in the 75 CRC patients by Kaplan-Meier analyses. As shown in Figure 2, *CHST10* and *EPDR1* hypermethylation was significantly correlated with a better prognosis (*CHST10*: *p* = 0.026; *EPDR1*: *p* = 0.018).

### 2.3. The Methylation Level of EPDR1 Is Correlated with Its mRNA Expression in CRC Tumor Tissues

**Figure 3:**
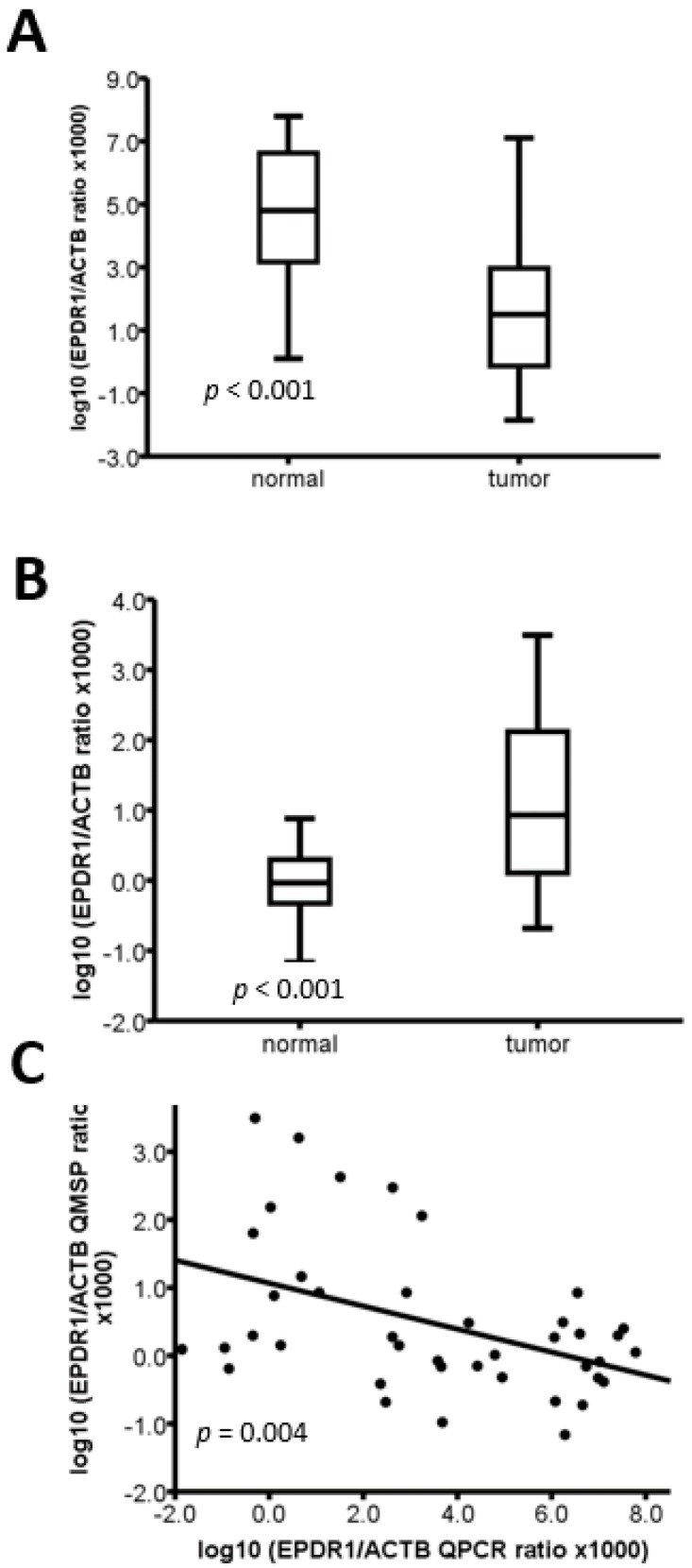
Methylation status of *EPDR1* and corresponding *EPDR1* mRNA levels in 23 paired CRC tissue specimens. (**A**) The mRNA level of *EPDR1* was analyzed by qRT-PCR. (**B**) The DNA methylation level of *EPDR1* was analyzed by Q-MSP. The mRNA and methylation levels of the *EPDR1* gene are expressed on the log_10_ scale. Box-and-whisker plots represent data with boxes ranging from the 25th to 75th percentile of the observed values, with the horizontal bar at the median value. The correlation between the qRT-PCR and Q-MSP results was assessed using linear regression.

To assess whether DNA methylation is associated with the expression of *EPDR1* and *CHST10*, qRT-PCR was performed to examine the mRNA expression of *EPDR1* and *CHST10* in 23 CRC tumor tissues and corresponding normal tissues. As shown in Figure 3A, we found that the level of *EPDR1* mRNA was significantly lower in tumor tissues than in corresponding normal tissues (*p* < 0.001), whereas the methylation level of *EPDR1* in 23 tumor tissues was significantly higher than that in corresponding normal tissues (*p* < 0.001; Figure 3B). Moreover, a negative correlation between the qRT-PCR and Q-MSP results was observed (*p* = 0.004), indicating that DNA methylation likely participates in regulating *EPDR1* expression (Figure 3C). Similarly, a negative correlation between mRNA expression and DNA methylation of the *EPDR1* gene in 195 colorectal adenocarcinoma patients was reported in The Cancer Genome Atlas (TCGA) dataset (Nature 2012) (Figure S2). Because the mRNA levels of *CHST10* were too low to be detected by qRT-PCR, we could not assess an inverse relationship between qRT-PCR and Q-MSP data.

### 2.4. EPDR1 Methylation Is Associated with BRAF and TGFβR2 Mutations in CRC Tumor Tissues

Because *APC*, *TP53*, *KRAS*, *BRAF*, *TGFβR2*, *PIK3CA*, and *SMAD4* are the most commonly mutated genes in CRC [35], we investigated the relationship between mutations in these genes and *EPDR1* methylation in 59 CRC tissues. Consistently, 17 of 27 cases (63%) in the hypermethylated *EPDR1* group (EPDR1-HYPER-M) were MSI positive, whereas 5 MSI-positive cases (15.6%) were observed for the hypomethylated *EPDR1* group (EPDR1-HYPO-M). Intriguingly, 16 of 17 cases were categorized as MSI with *MLH1* methylation. By contrast, 1 of 5 MSI cases with *MLH1* methylation was associated with *EPDR1* hypomethylation (EPDR1-HYPO-M). Figure S3 displays the mutation patterns of *APC*, *TP53*, *KRAS*, *BRAF*, *TGFβR2*, *PIK3CA*, and *SMAD4* in 27 EPDR1-HYPER-M patients and 32 EPDR1-HYPO-M patients. The EPDR1-HYPER-M group showed a higher mutation rate for *BRAF* and *TGFβR2* than the EPDR1-HYPO-M group. All 27 patients with hypermethylated *EPDR1* carried at least one mutation in these seven selected genes. However, nine of 32 cases (28.1%) in the EPDR1-HYPO-M group showed no mutation in these seven genes. Furthermore, differential mutation profiles between the EPDR1-HYPER-M and EPDR1-HYPO-M groups were noted. Among these seven genes, *EPDR1* methylation was significantly associated with *BRAF* (*p* < 0.001) and *TGFβR2* (*p* = 0.04) mutations in CRC tumor tissues (Table 3). All *BRAF* mutations in this study were the V600E substitution.

### 2.5. DNA Methylation Is Involved in the Regulation of EPDR1 Expression in CRC Cell Lines

**Figure 4:**
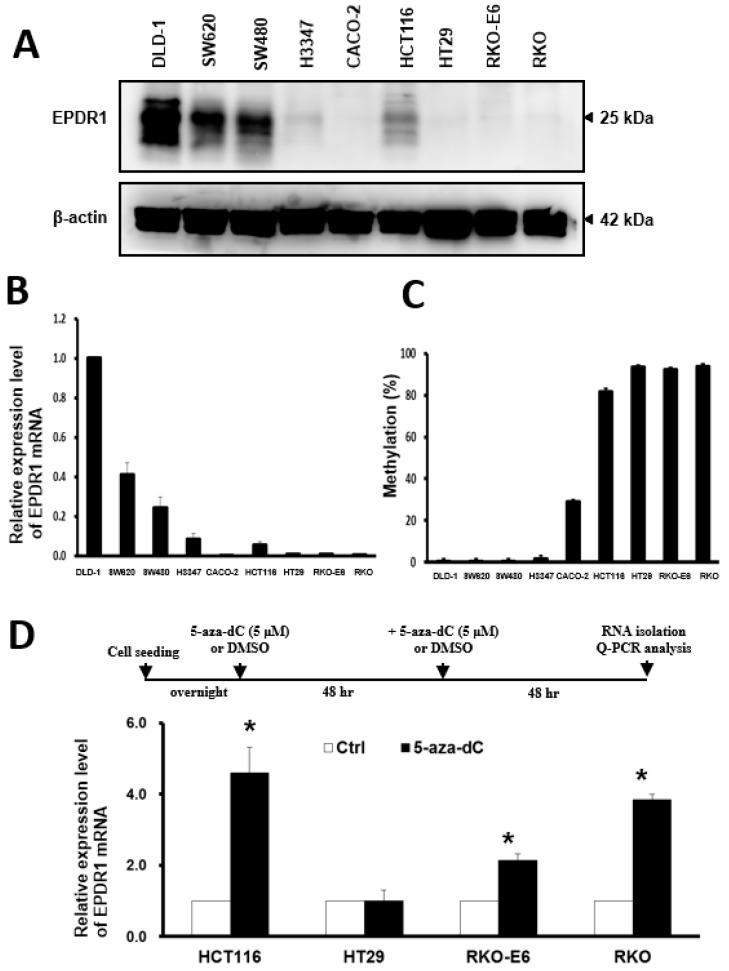
*EPDR1* expression and DNA methylation status in CRC cell lines. (**A**) The protein level of EPDR1 in CRC cell lines was examined by western blotting using β-actin as a loading control. (**B**) The mRNA level of *EPDR1* in CRC cell lines was examined by qRT-PCR using GAPDH as a loading control. The data are the means and SD of three independent experiments. The relative expression of *EPDR1* mRNA is expressed compared with that in DLD-1 cells. (**C**) The methylation levels of *EPDR1* in CRC cell lines were determined by bisulfite sequencing PCR (BSP) and were quantified as histograms. (**D**) The upper graph presents the detailed 5-aza-dC treatment schedule; (Bottom) Four CRC cell lines were treated with 5-aza-dC (5 μM) or dimethyl sulfoxide (DMSO; Ctrl) for 96 h and then were analyzed by qRT-PCR. The data are presented as the means and SD of three independent experiments. * *p* < 0.05 compared with Ctrl cells.

To evaluate whether epigenetic silencing contributes to a decrease in *EPDR1* expression, we performed qRT-PCR and western blotting to measure the expression of EPDR1 at both the mRNA and protein levels in nine CRC cell lines. As shown in Figure 4A,B, DLD-1 cells exhibited the highest levels of EPDR1 protein and mRNA, whereas SW480, SW620, H3347 and HCT116 cells expressed relatively lower levels of EPDR1. CACO-2, HT29, RKO, and RKO-E6 cells expressed limited or little amounts of EPDR1. Our results showed a satisfactory correlation between mRNA and protein expression among these cell lines. We further employed BSP to investigate whether the expression of *EPDR1* can be attributed to the methylation of regulatory elements in CRC cell lines. Thirty-one CpG sites were located within the +64 and +437 regions of the *EPDR1* gene (Figure S4A). As shown in Figure S4B, all 31 CpG sites were almost completely methylated in RKO, HT29, RKO-E6, and HCT116 cells. By contrast, the methylation levels in CACO-2, SW480, SW620, H3347, and DLD-1 cells were much lower. The quantitative BSP results for the nine CRC cell lines are shown in Figure 4C. These results reveal a close inverse association between promoter methylation and *EPDR1* expression at the mRNA and protein levels in CRC cell lines.

To verify the association between epigenetic aberrations and putative transcriptional inactivation of *EPDR1* gene expression, HCT116, HT29, RKO-E6, and RKO cells, which display high *EPDR1* promoter methylation levels, were treated with 5-aza-dC, a DNA demethylation reagent and assessed for the expression of *EPDR1* after 96 h of treatment by qRT-PCR (Figure 4D upper). As shown in Figure 4D, 5-azadC treatment significantly increased the *EPDR1* transcript abundance in HCT116, RKO-E6, and RKO cells but not in HT29 cells. As expected, the *EPDR1* transcript levels were not affected by 5-aza-dC treatment in *EPDR1*-hypomethylated cell lines, such as DLD-1, SW620, SW480, and H3347 cells (Figure S5).

### 2.6. EPDR1 Knockdown Suppresses CRC Cell Invasion

**Figure 5:**
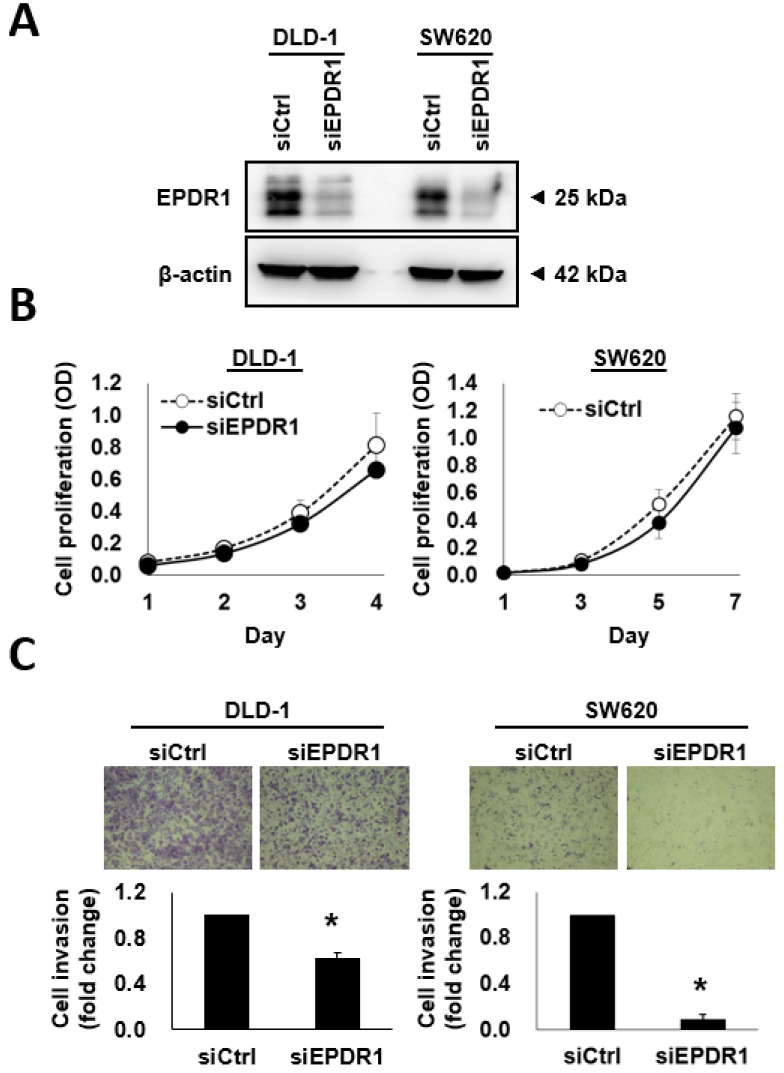
*EPDR1* knockdown suppresses invasion in CRC cells. Two CRC cell lines, DLD-1 and SW620, were transfected with either *EPDR1* small interfering RNA (siRNA, siEPDR1) or control siRNA (siCtrl). (**A**) The efficacy of *EPDR1* knockdown was examined by western blotting using β-actin as a loading control. (**B**) Cell proliferation was determined using the PrestoBlue cell viability reagent. The data are presented as the means and SD of three independent experiments. (**C**) The invasiveness of siEPDR1- and siCtrl-transfected cells was analyzed using Boyden chambers coated with a layer of Geltrex. The data are presented as the means and SD of three independent experiments. * *p* < 0.05 compared with siCtrl cells.

Ependymins are extracellular matrix proteins that inhibit cell adhesion. Because they possess anti-adhesive properties, EPDR1 might activate the detachment of cells from a solid tumor. Although few studies have reported EPDR1 expression in cancer cells, EPDR1 is known to be highly expressed in CRC [30]. Therefore, it is important to elucidate whether EPDR1 played a role in CRC cell invasion and metastasis. We performed western blotting to determine the efficacy of knockdown and found EPDR1 expression to be significantly decreased in DLD-1 and SW620 cells after 72–96 h of transient RNA interference (Figure 5A). Transient *EPDR1* knockdown did not affect the proliferative rate of CRC cells (Figure 5B) but did significantly suppress their invasion capacity (Figure 5C).

## 3. Discussion

Aberrant DNA methylation is associated with cancer progression, and studies on DNA methylation are likely to help in the identification of biomarkers clinically relevant to the process of tumorigenesis. Using the MethylCap-seq approach, Simmer et al. reported hypermethylation enrichment at gene promoter CpG islands in tumor samples, whereas hypomethylation was found throughout the genome [36]. In this study, we used a DNA methylation array to analyze differential DNA methylation patterns in CRC with MSI and found highly hypermethylation regions in the MSI-MLH1 methylated group compared with those in the MSI-MLH1 mut and MSS groups. MSI has been linked to hypermutation, hypermethylation, immune infiltration, and *BRAF* mutation [11]. Although it was demonstrated that most hypermethylated regions in the MSI group with *MLH1* promoter methylation were not associated with cancer progression, we found that *EPDR1* hypermethylation was associated with MSI, node status, tumor AJCC stage, and better prognosis in CRC patients. This is the first study to demonstrate the clinical prognostic values of *EPDR1* methylation in CRC. Although the sample size is limited in our study, our finding was supported by the data retrieved from TCGA (Figure S2). More patients will be enrolled to demonstrate that *EPDR1* methylation status could be a prognostic marker for CRC in future studies.

Epigenetic alterations have been linked to cancer-related gene transcriptional silencing. Based on the observed associations among qRT-PCR, Q-MSP, and western blotting results using CRC cell lines, we demonstrated that DNA methylation might play a critical role in regulating *EPDR1* expression in CRC cells. In the present study, HCT116, HT29, RKO, and RKO-E6 cells displayed *EPDR1* hypermethylation. Among them, RKO cells have been designated as a CIMP cell line; however, HT29 and HCT116 cells are thought to be non-CIMP cell lines [35]. Although *EPDR1* methylation might not be well associated with CIMP in established cell lines, our findings show that *EPDR1* hypermethylation is closely associated MSI with *MLH1* methylation in clinical specimens.

Approximately 10% of CRCs display *BRAF* mutations [37], mostly the V600E substitution, resulting in constitutive mitogen-activated protein kinase kinase (MEK) phosphorylation and BRAF signal transduction [38]. *BRAF*-mutant CRCs are generally characterized by MSI with MMR deficiency and very high mutation rates [38]. *BRAF* mutations confer a relatively poor survival, but this phenomenon is restricted to carcinomas not showing MSI [39]. Although we identified *EPDR1* as a hypermethylated gene in MSI patients with *MLH1* methylation, we discovered an association between *EPDR1* methylation and *BRAF* mutation. Therefore, the clinical implication of *EPDR1* hypermethylation with a high rate of *BRAF* mutation warrants further investigation.

Fang et al. demonstrated that the *BRAF* V600E mutation results in CIMP and transcriptional silencing of nearby genes through v-Maf avian musculoaponeurotic fibrosarcoma oncogene homolog G (MAFG), a transcriptional repressor [40]. Additionally, *BRAF* V600E reportedly increases BRAF/MEK/extracellular signal-regulated kinase (ERK) signaling and enhances MAFG levels, promoting the binding of DNA modifiers and modulating CpG island methylation [40]. Important mediators of DNA methylation and demethylation include DNA methyltransferases (DNMTs), methyl-CpG binding proteins (MeCPs), and ten-eleven translocation cytosine dioxygenases (TETs) [41]. It has been reported that, after H_2_O_2_ treatment, silencing protein complex containing sirtuin-1 (SIRT1), enhancer of zeste protein-2 (EZH2), DNA methyltransferase 1 (DNMT1), DNA methyltransferase 3 beta (DNMT3B), and H2A histone family member X (H2AFX) interact with the *EPDR1* gene (source: IntAct) [42]. Localization of this complex to the *EPDR1* gene may result in histone mark changes, reductions in nascent transcription, and increases in DNA methylation [42].

Although few studies to date have investigated *EPDR1* expression in cancer cells, it is highly expressed in CRC cells [36]. Nimmrich et al. found that the *EPDR1* transcript level is increased in cultured tumor cell lines (SW480 and HCT116) and in two of three analyzed CRC tissue specimens compared with that in a cultured normal cell line (NCM460) and in corresponding normal tissues [36]. By contrast, our results showed that the mRNA levels of *EPDR1* were lower in CRC tissue specimens than in corresponding non-cancerous tissues (*n* = 23). The difference between our findings and those in the previous study [36] might be due to the number of specimens analyzed. More CRC pairs will be examined to evaluate that expression of *EPDR1* during CRC pathogenesis in future studies.

5-aza-dC could reactivate *EDPR1* expression in several CRC cell lines, except HT29 cells, displaying a highly methylated *EPDR1* promoter. In addition to DNA methylation, histone acetylation is the other epigenetic modification that may modulate transcription. Several epigenetic regulatory genes can be activated by combined treatment with histone deacetylase inhibitor, such as trichostatin A, and 5-aza-dC [43]. However, the involvement of DNA methylation and histone acetylation in *EPDR1* expression in HT29 requires further elucidation.

Located at chromosome 7p14, the *EPDR1* gene encodes a protein comprising 224 amino acids with an ependymin domain that is a type II transmembrane protein similar to two families of cell adhesion molecules: protocadherins and ependymins [30]. Ependymin is a glycoprotein of the brain extracellular fluid that has been implicated in synaptic changes linked to the consolidation process of long-term memory formation [44]. It has been reported that ependymins are extracellular matrix proteins that inhibit cell adhesion. EPDR1, which has anti-adhesive properties, might promote the detachment of cells from a solid tumor. In this study, we demonstrated that EPDR1 was positively associated with the invasiveness of CRC cells.

DNA methylation of metastasis-related genes is a promising biomarker for CRC prognosis. Among metastasis-related genes, *p16INK4a* promoter methylation is mainly associated with a metastogenic phenotype of primary CRCs [45]. *Vimentin* gene methylation is also a potential prognostic marker for advanced CRC [46]. Alternatively, the promoter hypomethylation of *PGP9.5* is associated with invasion activity of CRC [47]. *PGP9.5* is therefore an invasive marker for CRC [48]. In this study, we found that *EPDR1* knockdown did not affect the proliferative rate of CRC cells but did significantly suppress their invasion capacity. Clinically, *EPDR1* hypermethylation was significantly correlated with node negativity and good prognosis. These results suggest that *EPDR1* hypermethylation may prevent CRC metastasis and serve as a prognostic marker.

## 4. Materials and Methods

### 4.1. Patient and Tumor Samples

This study included 75 CRC patients who were surgically treated at the Taipei Veteran General Hospital from 2000 to 2010. After approval by the Institutional Review Board at Taipei Veteran General Hospital (the IRB number is 201009003IC), CRC samples from this study were collected from the Biobank (Taipei Veterans General Hospital). Clinical information, including age, sex, personal and family medical history, location of tumor, TNM stage, differentiation, pathological prognostic features, and follow-up conditions, was retrieved from the hospital database. The 75 CRC patients were divided into two groups. MSS and MSI, based on MSI analysis. MSI patients were further divided into two groups, MSI with a methylated *MLH1* promoter (MSI-MLH1 methylated) and MSI with *MMR* mutation (MSI-MLH1 mut), based on the results of mutation analyses and methylation analyses of the *MLH1* gene. Three CRC tissues of each group were randomly selected for DNA pooling. All 75 tumor tissues and the corresponding normal tissues from patients with CRC were collected for Q-MSP. Among 75 patients, 23 CRC tissues and paired adjacent normal tissues were collected for qRT-PCR. Fifty-nine of 75 CRC samples were obtained for MassArray-based mutation characterization.

### 4.2. DNA Extraction from Tumor Samples

High-molecular-weight genomic DNA from CRC tumor samples and corresponding normal tissue samples was purified using the QIAamp Tissue kit (QIAGEN, Valencia, CA, USA) according to the manufacturer’s instructions. The yield and purity were determined using a Nanodrop 1000 Spectrophotometer (Thermo Fisher Scientific, Waltham, MA, USA).

### 4.3. MSI Analysis

MSI was characterized via the assessment of markers consisting of three dinucleotide repeats (D2S123, D5S346, D17S250) and two mononucleotide repeats (BAT26, BAT25) [49]. The primer sequences of five reference microsatellite markers were obtained from GenBank (www.gdb.org). Samples with more than two markers were defined as having MSI, and those patients with 0–1 MSI markers were considered as MSS.

### 4.4. Mutation Analysis of MLH1

DNA obtained from CRC tissues was amplified and sequenced with primers used in a previous study [50]. Sequencing of the *MLH1* gene covered its exons and intronic regions adjacent to all splice sites. The extracted DNA was amplified by polymerase chain reaction (PCR) in a DNA thermocycler. The PCR product produced was then sequenced. Each sample was sequenced on both sense and antisense strands. Each mutation was confirmed by a second sequencing on new PCR products.

### 4.5. Methylation Analysis of MLH1

Methylation of the *MLH1* promoter was determined by methylation-specific PCR. DNA was modified by sodium bisulfite and then was amplified with different methylated and unmethylated primers [51].

### 4.6. Genome-Wide DNA Methylation Profiling

A 0.5-μg sample of pooled DNA obtained from three early-staged CRC tissues was treated with sodium bisulfite using the EZ DNA Methylation-Gold Kit (Zymo Research, Orange, CA, USA) according to the manufacturer’s protocol. Methylation profile changes in these three groups were evaluated using the Infinium Methylation 27K BeadChip assay (Illumina), and CpG loci were validated using Illumina BeadStudio Software (Genetech Biotech, Taipei, Taiwan).

### 4.7. Quantitative Methylation-Specific PCR (Q-MSP)

After sodium bisulfite conversion, methylation analysis was performed using the Taqman PCR reaction-based MethyLight assay [52]. Primers and probes were designed for *EGF*, *CHST10*, *EPDR1*, *BST2*, and *RAC3*, as summarized in Supplementary Table S1. Primers and probes were designed to cover the same genomic region as found in the Infinium assay. β-Actin (*ACTB*) was amplified as a DNA loading control. Normal leukocyte DNA and in vitro-methylated leukocyte DNA served as negative and positive controls, respectively. PCR amplification was performed as previously described [53]. The relative level of methylated DNA for each gene in each sample was determined as a ratio of methylation-specific PCR-amplified DNA to *ACTB* DNA and then was multiplied by 1000 for easier tabulation. All values of methylation levels are presented on base 10 logarithmic scales. To compare the methylations levels of these five genes in tumor and corresponding normal tissues, we validated the cutoff values as follows: for *EGF*, tumor value/normal value > 1.5; for *CHST10*, log (tumor methylated value) > 2.5; for *EPDR1*, log (tumor methylated value) > 0.5; for *BST2*, tumor value/normal value > 0.5; for *RAC3*, tumor methylated value > 0.

### 4.8. Quantitative Real-Time Reverse Transcription PCR (qRT-PCR)

Total RNA was isolated using the TRI reagent (Molecular Research Center, Cincinnati, OH, USA) and was converted into first-strand cDNA using an oligo (dT) primer and the AMV reverse transcriptase system (Roche Diagnostics, Penzberg, Germany). qRT-PCR was performed using a LightCycler 480 system (Roche Diagnostics). Thermocycling was carried out in a final volume of 10 μL containing 3 μL of cDNA sample, 200 nM of each primer, and 5 μL of SYBR green I master mix (Roche Diagnostics). Relative differences in the expression level between genes were expressed using cycle threshold (Ct) values as follows: the Ct value of the gene of interest was first normalized to that of *GAPDH* in the same sample; the difference between the treatment and control group was then calculated, and it was expressed as an increase or decrease in the cycle number compared with that of the control.

### 4.9. MassArray-Based Mutation Characterization

The MassDetect CRC panel (v2.0), enabling identification of 139 mutations in 12 genes, was selected from hotspots found in a previous study and the COSMIC database [37,54]. PCR and extension primers for 139 mutations were designed using MassArray Assay Design 3.1 software (Sequenom, San Diego, CA, USA). The PCR products were spotted onto SpectroCHIP II arrays, and the DNA fragments were resolved using a MassArray Analyzer 4 System (Sequenom). Each spectrum was then analyzed by Type 4.0 software (Sequenom) to identify mutations. We defined 5% abnormal signals as a putative mutation.

### 4.10. Cell Lines and Cell Culture

Nine human CRC cell lines, DLD-1, H3347, SW480, SW620, Caco-2, HCT116, HT29, RKO, and RKO-E6, were used. DLD-1 (CCL-221), SW480 (CCL-228), SW620 (CCL-227), CACO-2 (HTB-37), HCT116 (CCL-247), HT29 (HTB-38), RKO (CRL-2577), and RKO-E6 (CRL-2578) cells were purchased from the American Type Culture Collection (ATCC). H3347 was kindly provided by Shih-Ching Chang (Taipei Veterans General Hospital, Taipei, Taiwan). All cell lines were cultured in Dulbecco’s Modified Eagle’s Medium (DMEM) (Thermo Fisher Scientific) with 10% fetal blood serum (FBS), L-glutamine, 100 U/mL of penicillin and 100 µg/mL of streptomycin at 37 °C in humidified air with 5% CO_2_.

### 4.11. Bisulfite Sequencing PCR (BSP)

BSP was performed as described previously [55]. Briefly, bisulfite-modified DNA was prepared and used as the template for amplification of the *EPDR1* gene promoter using AmpliTaq Gold^®^DNA Polymerase (Applied Biosystems, Carlsbad, CA, USA). The primers used for BSP are listed in Supplementary Table S2. The PCR products were subcloned into the TA cloning vector (Bioman Scientific, Taipei, Taiwan) and transformed into DH5α competent cells (Bioman Scientific). The plasmids were purified and sequenced. For all BSP assays, 15–20 independent clones for each CRC cell line were isolated and sequenced.

### 4.12. Western Blotting

Western blotting was performed as previously described [55,56]. Briefly, equal amounts of protein were electrophoretically separated by sodium dodecyl sulfate-polyacrylamide gel electrophoresis (SDS-PAGE) and were electrotransferred onto polyvinylidene fluoride (PVDF) membranes. The membranes were blocked and incubated overnight with primary antibodies against human EPDR1 (sc-81820; Santa Cruz Biotechnology, Santa Cruz, CA, USA). β-Actin (#ab6276; Abcam, Cambridge, UK) was used as a loading control.

### 4.13. 5-Aza-2’-Deoxycytidine (5-aza-dC) Treatment

CRC cells were seeded in 100-mm culture dishes. After incubation overnight, the culture medium was replaced with fresh medium containing 5 μM 5-aza-dC (Sigma-Aldrich, St. Louis, MO, USA) or dimethyl sulfoxide (DMSO), followed by incubation for 96 h. The culture medium was carefully replaced every 2 days. At the end of treatment, the cells were collected for qRT-PCR assays.

### 4.14. siRNA Transfection

DLD-1 and SW620 cells in 6-well plates were transfected with siRNA using the Lipofectamine 3000 transfection reagent (Invitrogen, Carlsbad, CA, USA) according to the manufacturer’s instructions. Validated double-stranded siRNAs for *EPDR1* (stB0005769A) or non-target control siRNAs (siN05815122147) (One Array, Hsinchu, Taiwan) were mixed with the transfection reagent and then were added to the cell culture. After 72 h, the cells were harvested for subsequent proliferation and invasion assays. Cell proliferation was determined using the PrestoBlue cell viability reagent (Thermo Fisher Scientific) according to the manufacturer’s protocol. EPDR1 expression levels in the siRNA-transfected cells were examined by western blotting.

### 4.15. Invasion Assay

The invasion assay was performed as previously described [56]. Briefly, an upper chamber containing a polycarbonate filter (8-μm pore size; Corning, Lowell, MA, USA) was coated with Geltrex (Life Technologies, Carlsbad, CA, USA); the lower chamber contained 700 μL of 10% FBS growth medium. In total, 1 × 10^5^ cells in 500 µL of 1% FBS growth medium were plated in the upper chamber and were allowed to move overnight toward the growth medium in the lower chamber. The invasive cells were fixed with 100% cold ethanol and were stained with Giemsa stain (Sigma-Aldrich) for 30 min.

### 4.16. Statistical Analysis

The distribution of each clinicopathological variable was compared using the two-tailed Fisher’s exact test and chi-squared test. Numerical values were compared using Student’s *t* test. The data are expressed as the means ± standard deviation (SD). Statistical significance was defined as *p* < 0.05. The Kaplan–Meier method using the log-rank test was used to estimate overall survival (SPSS software 17.0; SPSS Inc., Chicago, IL, USA).

## 5. Conclusions

The present study demonstrated that *EPDR1* hypermethylation is significantly correlated with a negative node status, a lower tumor stage, BRAF and TGFβR2 mutations and better prognosis in CRC patients. We further showed that DNA methylation modulated *EPDR1* expression in CRC cells. The biological functions and involvement of *EPDR1* in CRC progression may be related to CRC cell invasion. Understanding the biological functions and regulatory mechanisms of *EPDR1* in CRC progression may provide new insight into the development of novel strategies for CRC treatment.

## Figures and Tables

**Table 1:** Clinicopathological features of 75 patients with colorectal cancer (CRC).

	*n*	MSS *	MSI ^#^	*p* Value
Gender				
Male	42	28 (66.7)	14 (33.3)	0.004
Female	33	11 (33.3)	22 (66.7)	
Age		76.9 ± 3.6	62.9 ± 14.3	<0.001
Stage				
I–II	44	19 (43.2)	25 (56.8)	0.069
III–IV	31	20 (64.5)	11 (35.5)	
Differentiation				
Well-moderate	63	38 (60.3)	25 (39.7)	0.001
Poor	12	1 (8.3)	11 (91.7)	
Location				
Proximal colon	27	5 (18.5)	22 (81.5)	<0.001
Distal colon	48	34 (70.8)	14 (29.2)	
Histology				
Adenocarcinoma	68	36 (52.9)	32 (47.1)	0.704
Mucinous	7	3 (42.9)	4 (57.1)	

* MSS: microsatellite-stable. ^#^ MSI: microsatellite instability.

**Table 2:** The association of methylation of *EGF*, *CHST10*, *EPDR1*, *BST2*, and *RAC3* with clinicopathologic features in CRC patients.

	*EGF*			*CHST10*			*EPDR1*			*BST2*			*RAC3*		
	Hypo-M	Hyper-M	*p*	Hypo-M	Hyper-M	*p*	Hypo-M	Hyper-M	*p*	Hypo-M	Hyper-M	*p*	Hypo-M	Hyper-M	*p*
Differentiation															
Well-moderate	33 (52.4)	30 (47.6)		29 (46.0)	34 (56.0)		33 (52.4)	30 (47.6)		29 (46.0)	34 (54.0)		32 (52.5)	29 (47.5)	
Poor	5 (41.7)	7 (58.3)	0.496	3 (25.0)	9 (75.0)	0.177	5 (41.7)	7 (58.3)	0.496	8 (66.7)	4 (33.3)	0.190	9 (75.0)	3 (25.0)	0.150
Invasion depth															
T1/T2	5 (38.5)	8 (61.5)		5 (38.5)	8 (61.5)		6 (46.2)	7 (53.8)		6 (46.2)	7 (53.8)		6 (54.5)	5 (45.5)	
T3/T4	33 (53.2)	29 (46.8)	0.333	27 (43.5)	35 (56.5)	0.736	32 (51.6)	30 (48.4)	0.720	31 (50.0)	31 (50.0)	0.801	35 (56.5)	27 (43.5)	1.00
Node stage															
Negative	23 (52.3)	21 (47.7)		16 (36.4)	28 (63.6)		18 (40.9)	26 (59.1)		22 (50.0)	22 (50.0)		26 (61.9)	16 (38.1)	
Positive	15 (48.4)	16 (51.6)	0.740	16 (51.6)	15 (48.4)	0.189	20 (64.5)	11 (35.5)	0.044	15 (48.4)	16 (51.6)	0.891	15 (48.4)	16 (51.6)	0.250
AJCC stage															
I–II	23 (52.3)	21 (47.7)		16 (36.4)	28 (63.6)		18 (40.9)	26 (59.1)		22 (50.0)	22 (50.0)		26 (61.9)	16 (38.1)	
III–IV	15 (48.4)	16 (51.6)	0.740	16 (51.6)	15 (48.4)	0.189	20 (64.5)	11 (35.5)	0.044	15 (48.4)	16 (51.6)	0.891	15 (48.4)	16 (51.6)	0.250
Microsatellite status															
MSS	18 (46.2)	21 (53.8)		23 (59.0)	16 (41.0)		28 (71.8)	11 (28.2)		14 (35.9)	25 (64.1)		14 (35.9)	25 (64.1)	
MSI	20 (55.6)	16 (44.4)	0.416	9 (25.0)	27 (75.0)	0.003	10 (27.8)	26 (72.2)	<0.001	23 (63.9)	13 (36.1)	0.015	28 (77.8)	8 (22.2)	<0.001

*EGF*: epidermal growth factor; *CHST10*: carbohydrate sulfotransferase 10; *EPDR1*: ependymin related 1; *BST2*: bone marrow stromal antigen 2; *RAC3*: Rac family small GTPase 3.

**Table 3:** Mutation status of *APC*, *TP53*, *KRAS*, *BRAF*, *TGFβR2*, *PIK3CA* and *SMAD4* in 59 patients with CRC.

	*n*	EPDR1 HYPER-M ^#^ (*n* = 27)	EPDR1 HYPO-M (*n* = 32)	*p* Value
*APC*				
Wild type	41	20 (74.1)	21 (65.6)	0.483
Mutation	18	7 (25.9)	11 (34.4)	
*TP53*				
Wild type	46	22 (81.5)	24 (75.0)	0.550
Mutation	13	5 (18.5)	8 (25.0)	
*KRAS*				
Wild type	45	21 (77.8)	24 (75.0)	0.803
Mutation	14	6 (22.2)	8 (25.0)	
*BRAF*				
Wild type	49	17 (63.0)	32 (100.0)	<0.001
Mutation	10	10 (37.0)	0 (0.0)	
*TGFBR2*				
Wild type	52	21 (77.8)	31 (96.9)	0.040
Mutation	7	6 (22.2)	1 (3.1)	
*PIK3CA*				
Wild type	46	19 (70.4)	27 (84.4)	0.196
Mutation	13	8 (29.6)	5 (15.6)	
*SMAD4*				
Wild type	57	25 (92.6)	32(100.0)	0.205
Mutation	2	2 (7.4)	0 (0.0)	

^#^ The cutoff value of *EPDR1* was log (tumor methylated value) >0.5. *APC*: adenomatous polyposis coli; *TP53*: tumor protein 53; *SMAD4*: SMAD family member 4; *KRAS*: K-ras proto-oncogene; *PI3KCA*: phosphoinositide 3-kinase catalytic subunit-α.

## Supplementary Materials

The following are available online at http://www.mdpi.com/2072-6694/10/10/393/s1, Table S1: Q-MSP primer and probe; Table S2: Bisulfite-sequencing PCR (BSP) primer sequences for *EPDR1* gene; Figure S1: Differential DNA methylation patterns in microsatellite stability (MSS), microsatellite instability (MSI)-methylated hMLH1 and MSI-mutated MMR CRC; Figure S2: The figure generated using cBioportal (http://www.cbioportal.org/). Shown are selected genomic profiles including mutations, putative copy-number alterations from GISTIC and mRNA expression data based on mRNA expression z-scores (RNA Seq RPKM). *EPDR1* was entered under Gene Set. The plot indicating the correlation between *EPDR1* mRNA expression (horizontal axis) and DNA methylation (vertical axis) was downloaded for visualization. These data were download to assess *p* values using linear regression; Figure S3: Mutation patterns of seven genes in 59 CRC patients; Figure S4: Methylation status of the +64 to +437 region of *EPDR1* in CRC cell lines; Figure S5: Four CRC cell lines were treated with 5-aza-dC (5 μM) or DMSO (Ctrl) for 96 h and then were analyzed by qRT-PCR.
