# Supplementary material for: Prognostic Values of EPDR1 Hypermethylation and Its Inhibitory Function on Tumor Invasion in Colorectal Cancer
Source: Cancers (Basel). 2018 Oct 22;10(10):393. doi: 10.3390/cancers10100393 (PMC6211107; doi:10.3390/cancers10100393)
Supplement: Supplementary file 1 [file cancers-10-00393-s001.pdf]

Supplementary material

Prognostic Values of EPDR1 Hypermethylation and Its Inhibitory Function on Tumor Invasion in Colorectal Cancer

Chun-Ho Chu, Shih-Ching Chang, Hsiu-Hua Wang, Shung-Haur Yang, Kuo-Chu Lai and Te-Chang Lee

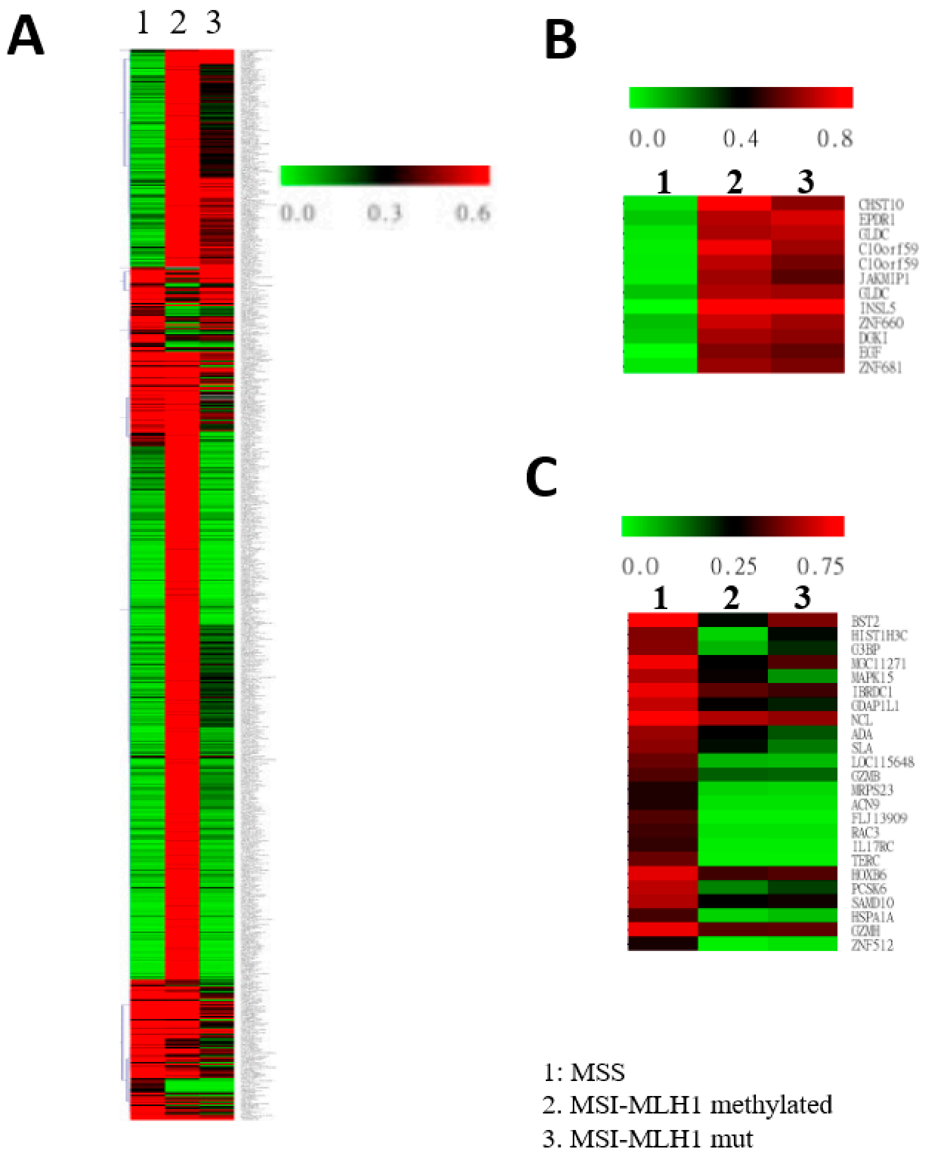

Figure S1. Differential DNA methylation patterns in microsatellite stability (MSS), microsatellite instability (MSI)-methylated hMLH1 and MSI-mutated MMR CRC. (**A**) Heatmaps showing 650 selected genes from the MSI group with *MLH1* promoter methylation or MMR mutation had an absolute beta difference ($\Delta\beta$) value of more than 0.5 (hypermethylation) or less than −0.25 (hypomethylation). Red represents increased hypermethylation; green indicates hypomethylation. (**B**) Ten hypermethylated genes in the MSI group and (**C**) 24 hypomethylated genes in both MSI groups were observed compared to the MSS group.

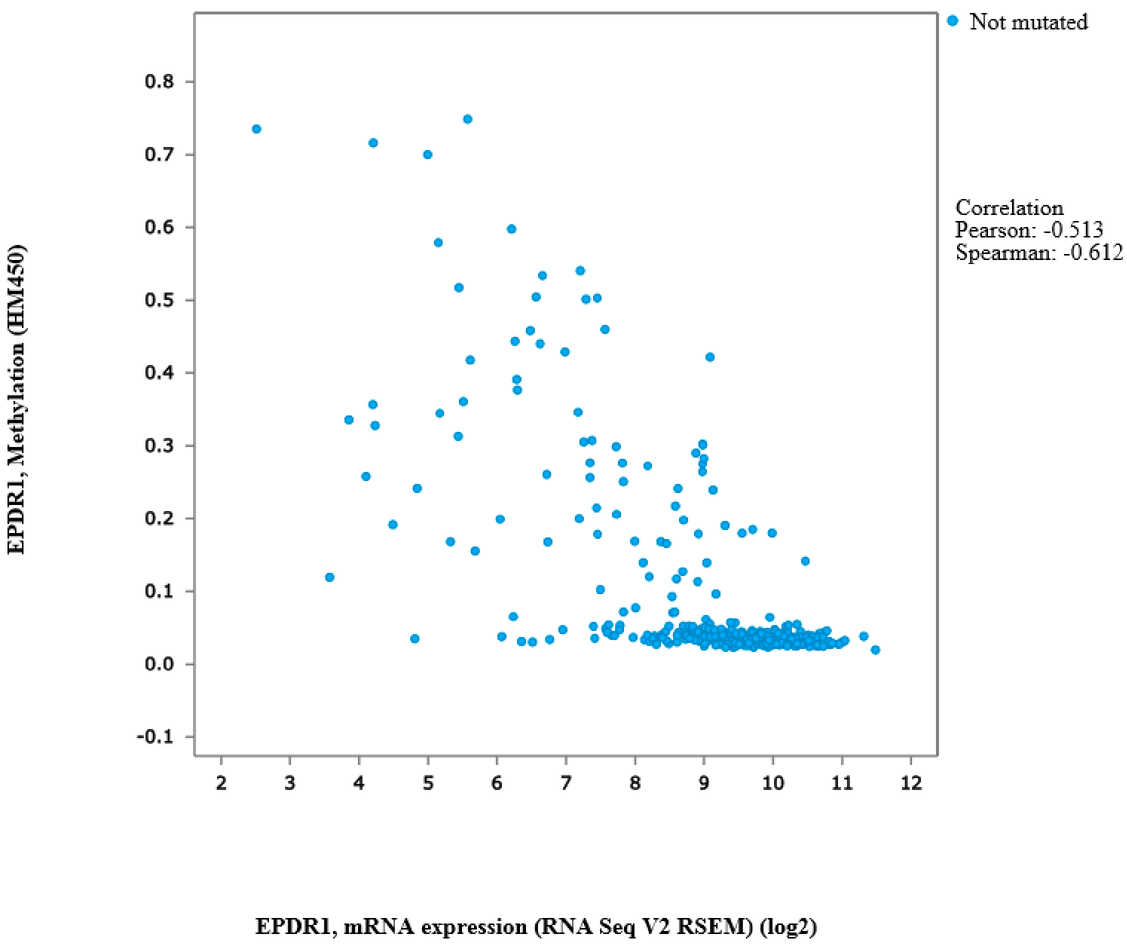

Figure S2. The figure generated using cBioportal (http://www.cbioportal.org/). Shown are selected genomic profiles including mutations, putative copy-number alterations from GISTIC and mRNA expression data based on mRNA expression z-scores (RNA Seq RPKM). *EPDR1* was entered under Gene Set. The plot indicating the correlation between *EPDR1* mRNA expression (horizontal axis) and DNA methylation (vertical axis) was downloaded for visualization. These data were downloaded to assess *p* values using linear regression.

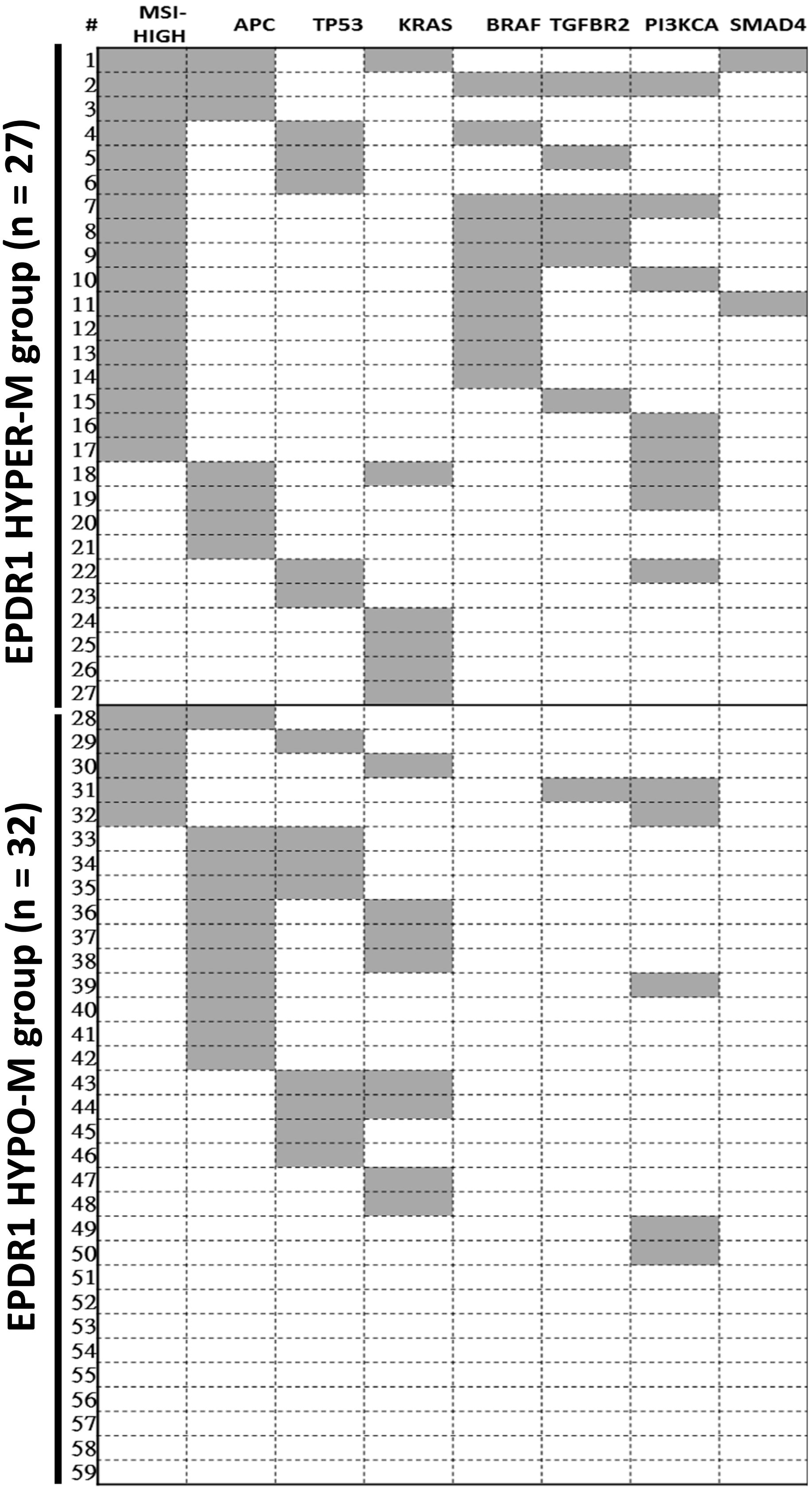

Figure S3. Mutation patterns of seven genes in 59 CRC patients. CRC patients were divided into two groups based on the methylation level of *EPDR1*: EPDR1-HYPER-M (*n* = 27) and EPDR1-HYPO-M (*n* = 32) groups. Columns show the results for each of the 59 cases analyzed. Gray boxes indicate cases involving mutation. The MSI status of each patient is listed in the first column.

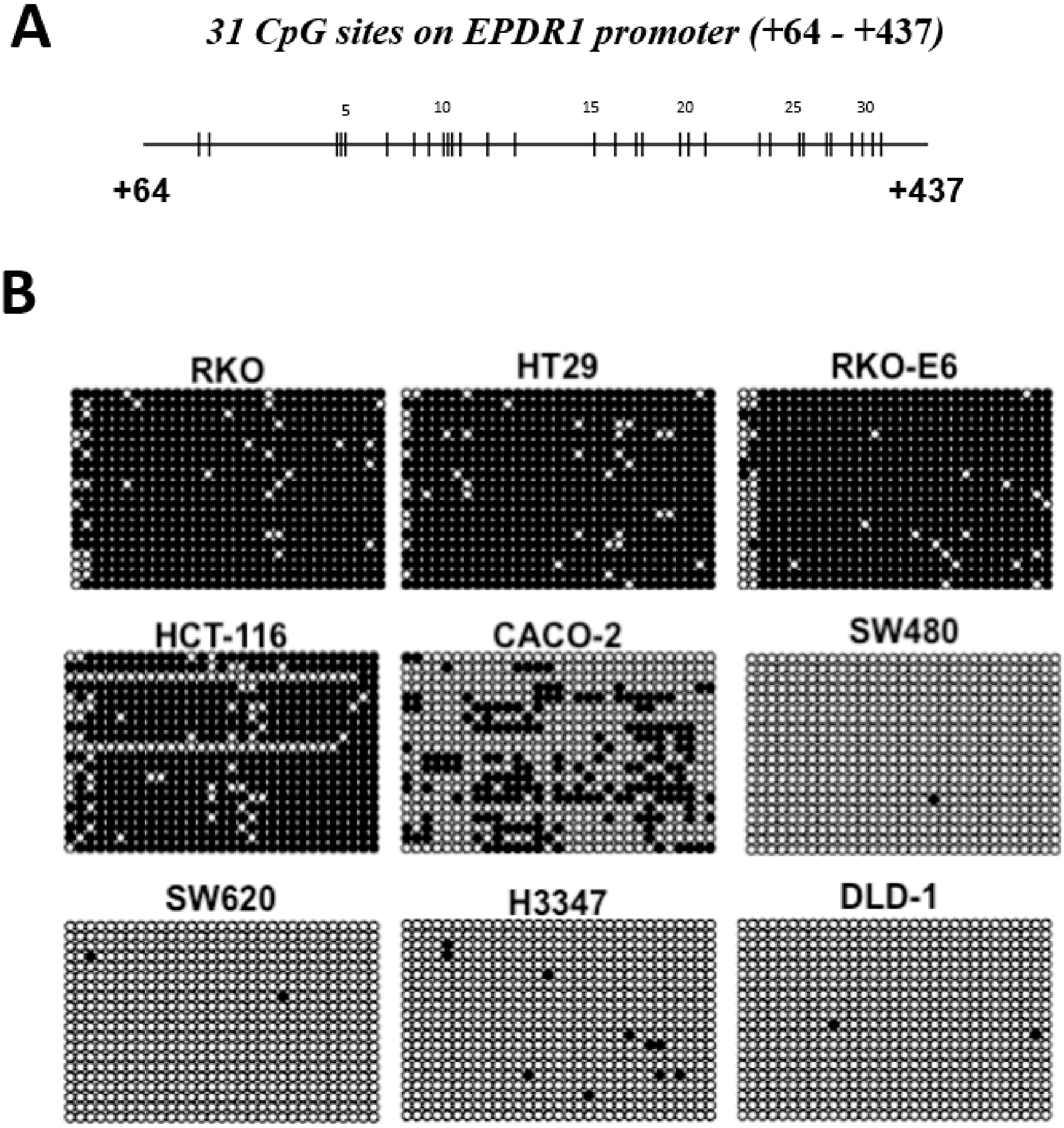

Figure S4. Methylation status of the +64 to +437 region of *EPDR1* in CRC cell lines. (**A**)The location of 31 CpG sites of the +64 to +437 region of *EPDR1* was analyzed by BSP. (**B**)The positions analyzed by BSP are indicated with black vertical bars. The overall methylation percentage indicates the total proportion of methylated CpGs in this region taking into account all sequenced alleles.

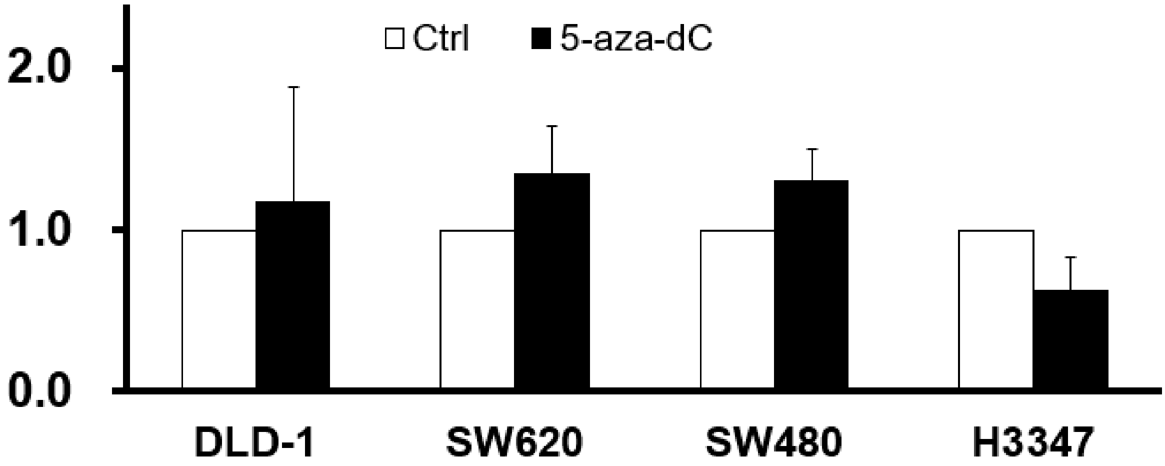

Figure S5. Four CRC cell lines were treated with 5-aza-dC (5 μM) or DMSO (Ctrl) for 96 h and then were analyzed by qRT-PCR. The data are presented as the means and SD of three independent experiments.

Table S1. Q-MSP primer and probe.

Gene Name	Forward 5'–3'	Reverse 5'–3'	Probe
EGF	AGTAGTTTTCGTTATTTATTATGAGTATTT	CACACAAAAAAACAAAAATAACTACCA	Fluorescein-SPC-TTTTGTTTAGGATATTTATGGTGAGTAGCGAGT-Phosphate
CHST10	GATTTGTTTCGGTCGTAGG	TCCAACGAATCGCGAAAAATA	Fluorescein-SPC-AGCGGTAGAAAGGGAGATTCGG-Phosphate
EPDR1	TCGCGCGATTTATTGGAG	ACCGAAAATATAATCTAAACCAAAATACT	Fluorescein-SPC-AGCGGTAGAGGTTATCGAAGGG-Phosphate
BST2	GGAGATTTGGATGGTATTTATTTCG	CCAAAATTCCTATCCCCAACA	Fluorescein-SPC-AGACGGGGATAAGCGTTGTAAG-Phosphate
RAC3	CGTTACGTGGGAGTATATCGG	ACAAACGCGCTATATTTCCAA	Fluorescein-SPC-TTTTTCGTTTTTTGCGATGTTATCGTGAAC-Phosphate
ACTB	TGGTGATGGAGGAGGTTTAGTAAGT	AACCAATAAAACCTACTCCTCCCTTAA	Fluorescein-SPC-ACCACCACCCAACACACAATAACAAACACA-Phosphate

Table S2. BSP primer sequences for *EPDR1* gene.

Gene Name	Forward 5'–3'	Reverse 5'–3'
EPDR1	GTTGGTAGTAGTGAGTAGTGAA	CCAACAACCAAACACCCAAAA

© 2018 by the authors. Licensee MDPI, Basel, Switzerland. This article is an open access article distributed under the terms and conditions of the Creative Commons Attribution (CC BY) license (http://creativecommons.org/licenses/by/4.0/).
